# Immunogenicity of the Adjuvanted Recombinant Zoster Vaccine: Persistence and Anamnestic Response to Additional Doses Administered 10 Years After Primary Vaccination

**DOI:** 10.1093/infdis/jiaa300

**Published:** 2020-06-05

**Authors:** Andrew Hastie, Grégory Catteau, Adaora Enemuo, Tomas Mrkvan, Bruno Salaun, Stephanie Volpe, Jan Smetana, Lars Rombo, Tino Schwarz, Karlis Pauksens, Caroline Hervé, Adriana Bastidas, Anne Schuind

**Affiliations:** 1 GSK, Rockville, Maryland, USA; 2 GSK, Wavre, Belgium; 3 GSK, Rixensart, Belgium; 4 Faculty of Military Health Sciences, University of Defence, Hradec Kralove, Czech Republic; 5 Centre for Clinical Research, Eskilstuna, Sweden; 5a Uppsala University, Sweden; 6 Klinikum Wuerzburg Mitte, Standort Juliusspital, Wuerzburg, Germany; 7 Department of Infectious Diseases, Uppsala University Hospital, Uppsala, Sweden; 8 UCB Pharma, Braine-L’Alleud, Belgium; 9 Mithra Pharmaceuticals, Flémalle, Belgium

**Keywords:** herpes zoster, adjuvanted recombinant zoster vaccine, persistence of immune response, safety

## Abstract

**Background:**

The adjuvanted recombinant zoster vaccine (RZV) is highly immunogenic and efficacious in adults ≥50 years of age. We evaluated (1) long-term immunogenicity of an initial 2-dose RZV schedule, by following up adults vaccinated at ≥60 years of age and by modeling, and (2) immunogenicity of 2 additional doses administered 10 years after initial vaccination.

**Methods:**

Persistence of humoral and cell-mediated immune (CMI) responses to 2 initial RZV doses was assessed through 10 years after initial vaccination, and modeled through 20 years using a Piecewise, Power law and Fraser model. The immunogenicity and safety of 2 additional RZV doses were also evaluated.

**Results:**

Seventy adults were enrolled. Ten years after initial vaccination, humoral and CMI responses were approximately 6-fold and 3.5-fold, respectively, above those before the initial vaccination levels. Predicted immune persistence through 20 years after initial vaccination was similar across the 3 models. Sixty-two participants (mean age [standard deviation], 82.6 [4.4] years) received ≥1 additional RZV dose. Strong anamnestic humoral and CMI responses were elicited by 1 additional dose, without further increases after a second additional dose.

**Conclusions:**

Immune responses to an initial 2-dose RZV course persisted for many years in older adults. Strong anamnestic immune responses can be induced by additional dosing 10 years after the initial 2-dose course.

**Clinical Trials Registration:**

NCT02735915.

## Teaser

Long-term immunogenicity data and mathematical models suggest that immune responses to a 2-dose recombinant zoster vaccine course persist for many years in older adults. Strong anamnestic immune responses can be induced by additional dosing 10 years after the initial course.

Herpes zoster (HZ) results from reactivation of latent varicella-zoster virus (VZV), and occurs most frequently in older adults [1, 2]. In 2 large phase III trials (ZOE-50 and ZOE-70 [3, 4]), an adjuvanted recombinant zoster vaccine (RZV [Shingrix; GSK]), consisting of the VZV glycoprotein E (gE) antigen and the AS01_B_ adjuvant system, demonstrated ≥90% efficacy against HZ in all age groups among adults ≥50 years of age, which was maintained over a follow-up period of approximately 4 years [3, 4]. In the same population, humoral and cell-mediated immune (CMI) responses to RZV were assessed up to 2 years after vaccination and shown to persist through the last assessment [5]. Since 2017, RZV has received licensure for the prevention of HZ in adults aged ≥50 years of age in several countries.

**Figure 1.**
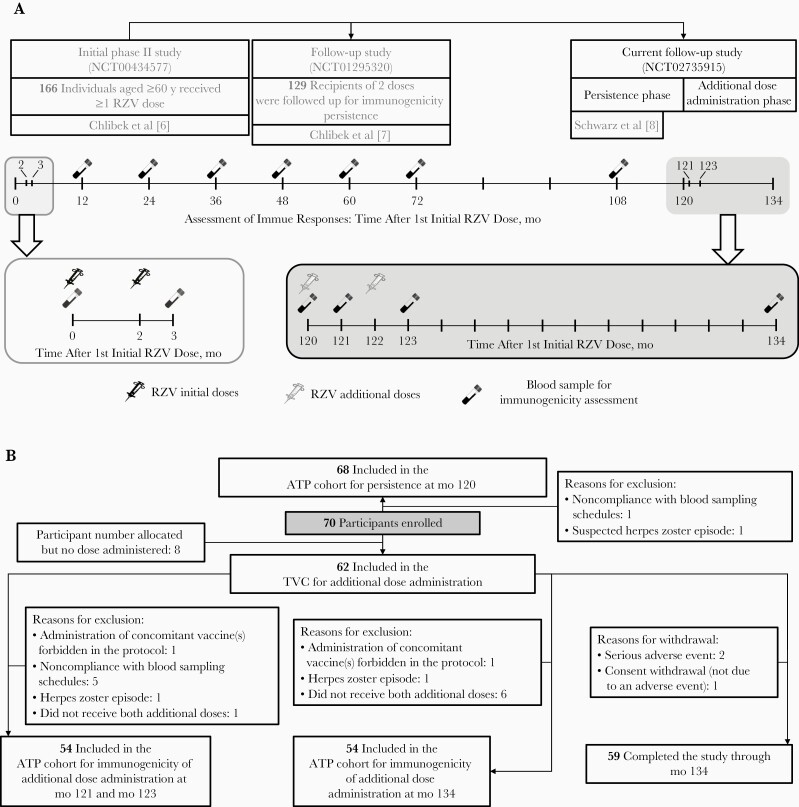
Overview of clinical trials (*A*) [6–8] and flow diagram of study participants (*B*). Abbreviations: ATP, according-to-protocol; mo, month; RZV, adjuvanted recombinant zoster vaccine; TVC, total vaccinated cohort.

In a phase II dose-ranging study, safety and immunogenicity of different investigational unadjuvanted or AS01_B_-adjuvanted gE vaccine formulations, including the licensed RZV formulation, were evaluated up to 3 years after vaccination in adults ≥60 years of age (NCT00434577; Figure 1A) [6]. Study participants who received 2 doses of the licensed RZV formulation were followed up for immunogenicity persistence at 4, 5, and 6 years (NCT01295320) and again at 9 and 10 years (NCT02735915) after initial RZV vaccination (Figure 1A) [7, 8]. We reported previously on the persistence of immune responses up to 9 years after vaccination; in addition, mathematical modeling predicted persistence of immune responses up to 15 years after vaccination [8].

In the current article, we present persistence data for the immune responses up to 10 years after initial vaccination, along with an updated prediction of persistence by mathematical modeling up to 20 years after initial vaccination. In addition, the immune memory responses and safety of 2 additional RZV doses were evaluated 10 years after the initial 2-dose vaccination schedule.

## METHODS

### Study Design

This was a single-arm, open-label, phase IIIB, long-term follow-up study of the initial phase II trial (NCT00434577) and was conducted between April 2016 and October 2018 in the Czech Republic (1 center), Germany (4 centers), and Sweden (2 centers). It included evaluation of immune responses at 9 and 10 years after initial RZV doses and modeling of 20-year persistence of immune responses using immunogenicity data acquired through 10 years after initial vaccination. This study also aimed to assess whether antigen challenge through additional dosing elicits an anamnestic response.

All participants provided written informed consent at enrollment. The study protocol was reviewed and approved by independent ethics committees or institutional review boards. The study was conducted in accordance with the Declaration of Helsinki and the principles of Good Clinical Practice. The study is registered at ClinicalTrials.gov (NCT02735915). Anonymized individual participant data and study documents can be requested for further research at www.clinicalstudydatarequest.com (study 204926).

### Study Participants

The initial phase II trial (NCT00434577) enrolled adults ≥60 years of age, according to the previously described inclusion or exclusion criteria [6]. In this phase IIIB extension study, individuals who participated in the initial phase II trial could be enrolled if they completed a 2-dose vaccination course with the licensed RZV formulation during that trial. From the single site in the Netherlands that participated in the initial study, only 3 participants were eligible for participation and were not invited to participate for logistical reasons [8]. A complete list of inclusion and exclusion criteria is presented in Supplementary Text 1.

### Study Vaccine

Two additional RZV doses were administered intramuscularly in the deltoid muscle of the nondominant arm at months 120 and 122 after the first dose of the initial 2-dose vaccination course. RZV consists of 50 μg of VZV gE antigen and the GSK proprietary AS01_B_ adjuvant system, containing 50 μg of MPL and 50 μg of QS-21 in a liposomal formaulation, per 0.5 mL of reconstituted vaccine.

### Outcomes and Assessments

#### Objectives

The primary objective of this study was to evaluate persistence of humoral and CMI responses to RZV at months 108 and 120 after first initial vaccine dose. To provide a full picture of the immune response’s kinetics over 10 years after an initial 2-dose vaccination course, immunogenicity results from the initial phase II study [6] and the phase II follow-up study [7] are also included herein. Safety in terms of vaccine-related serious adverse events (SAEs) was also evaluated between months 108 and 120.

The secondary objectives were evaluation of humoral and CMI responses to RZV at 1 month after dose 1, and at 1 and 12 months after dose 2 in the additional vaccination series. Reactogenicity and safety of the 2 additional RZV doses were also evaluated as a secondary objective. Based on the immunogenicity data collected through 10 years after initial vaccination, mathematical modeling of immunological persistence was performed as an exploratory objective.

#### Assessment of Immunogenicity

In this phase IIIB study, humoral and CMI responses were assessed at months 108 (9 years after initial dose 1), 120 (10 years after initial dose 1), 121 (1 month after additional dose 1), 123 (1 month after additional dose 2), and 134 (12 months after additional dose 2) after the first dose of the initial vaccination (Figure 1A). Anti-gE antibody concentrations were measured by anti-gE enzyme-linked immunosorbent assay with a technical cutoff assay quantification of 97 mIU/mL [5]. Frequencies of gE-specific CD4^2+^ T cells (CD4^+^ T cells expressing ≥2 of the 4 activation markers assessed: interferon γ, interleukin 2, tumor necrosis factor α, and CD40 ligand) were measured by means of intracellular cytokine staining and detection by flow cytometry after in vitro stimulation with a pool of peptides covering the gE ectodomain [5].

#### Assessment of Reactogenicity and Safety

For the initial vaccination course, we collected the occurrence of SAEs that were vaccine related or that led to death or withdrawal and the occurrence of suspected HZ cases documented between months 108 and 120 after initial vaccination. For the additional doses, diary cards were provided to the participants at each vaccination visit to record solicited local (injection-site pain, redness, and swelling) and general (fatigue, fever [body temperature ≥37.5°C], gastrointestinal symptoms [nausea, vomiting, diarrhea, and/or abdominal pain], headache, myalgia, and shivering) adverse events (AEs) during the 7 days after each vaccination, and unsolicited AEs during the 30 days after each vaccination. Grading of these events is described in Supplementary Text 2.

SAEs and potential immune-mediated diseases were recorded from first additional dose up to 12 months after the last additional dose. AEs or SAEs leading to withdrawal from the study and intercurrent medical conditions (including conditions that may confound interpretation of immunogenicity results, such as suspected HZ) were recorded from consent to participate in this phase IIIB study through 12 months after the last additional dose. All solicited local AEs were considered to be causally related to vaccination. For other AEs, the relationship between the study vaccine and the occurrence of the AE was assessed by the investigator.

### Statistical Analyses

All end points were descriptive and no formal sample size calculations were performed. The safety analysis for the initial vaccination course was performed on all participants enrolled in this phase IIIB study. Persistence of immune responses to the initial 2-dose RZV course was evaluated in the according-to-protocol (ATP) cohort for persistence, which included all evaluable participants from the ATP cohort for immunogenicity in the initial phase II trial who complied with the protocol and had immunogenicity results available at the considered time point. Modeling of humoral and CMI response persistence after initial vaccination was performed on the modeling cohort, which included participants of the initial phase II study who received 2 RZV doses, complied with its protocol, and who consented to participate in the long-term follow-up study that evaluated immune responses through 6 years after initial vaccination [7]. 

The safety of the additional RZV doses was analyzed in the total vaccinated cohort for additional dose administration, including all participants who received ≥1 additional RZV dose. Immunogenicity of the additional RZV doses was evaluated in the ATP cohort for immunogenicity of additional dose administration, that is, in participants who complied with the protocol and had immunogenicity results available at the considered time point. Individuals in whom an intercurrent medical conditions developed were excluded from subsequent ATP immunogenicity assessments.

Anti-gE antibody geometric mean concentrations (GMCs) were determined along with their exact 2-sided 95% confidence intervals (CIs). For gE-specific CD4^2+^ T cells, mean frequencies were calculated along with their standard deviation (SD). The frequency of gE-specific CD4^2+^ T cells was calculated as the difference between the frequency of CD4^2+^ T cells stimulated in vitro with gE peptides and those stimulated with culture medium alone, and the results were expressed as the number of CD4^2+^ T cells per 10^6^ total T cells.

Modeling of anti-gE antibody GMCs and geometric mean CD4^2+^ T-cell frequencies up to 20 years after initial vaccination was performed using 3 approaches: the Piecewise linear mixed model (assuming that the immune response declines linearly over time at a rate varying between nonoverlapping time intervals), the Power law model (assuming that the biological dynamics of the immune response declines over time following a logarithmic function), and the Fraser model (a modified Power law model including a constant in the logarithmic function) for repeated measurements (all immunogenicity data available; from months 0, 3, 12, 24, 36, 48, 60, 72, 108, and 120 [6–8]). All 3 models have been described in detail elsewhere [8]. Statistical analyses were performed using the SAS Drug Development software.

## RESULTS

### Study Participants

Approximately 6 years after initial RZV vaccination [6], 70 participants (mean age [SD] at first initial vaccination, 72.3 [4.3] years) were enrolled in this extension study [8], of whom 68 (mean age at first initial vaccination, 72.2 [4.3] years) were included in the ATP cohort for persistence at month 120. Sixty-two participants received ≥1 additional dose and were included in the total vaccinated cohort for additional dose administration. The mean age (SD) at first additional dose administration was 82.6 (4.4) years (Figure 1B and Table 1).

**Table 1.** Summary of Demographic Characteristics

Characteristic	ATP Cohort for Persistence (n = 68)	TVC for Additional Dose Administration (n = 62)
Age, mean (SD), y	72.2 (4.3)	82.6 (4.4)^a^
Sex, no. (%)		
Female	42 (61.8)	40 (64.5)
Male	26 (38.2)	22 (35.5)
Age group at 1st initial dose, no.		
60–69 y	13	11
≥70 y	55	51
Ethnicity or geographic ancestry, no. (%)		
Not American Hispanic or Latino	68 (100)	62 (100)
White/European ancestry	68 (100)	62 (100)

Abbreviations: ATP, according-to-protocol; SD, standard deviation; TVC, total vaccinated cohort; y, years.

^a^Note: All parameters refer to the first initial vaccination dose, except age in the TVC for additional dose administration, which refers to first dose of the additional 2-dose schedule

### Immunogenicity Persistence After Initial Vaccination

**Figure 2.**
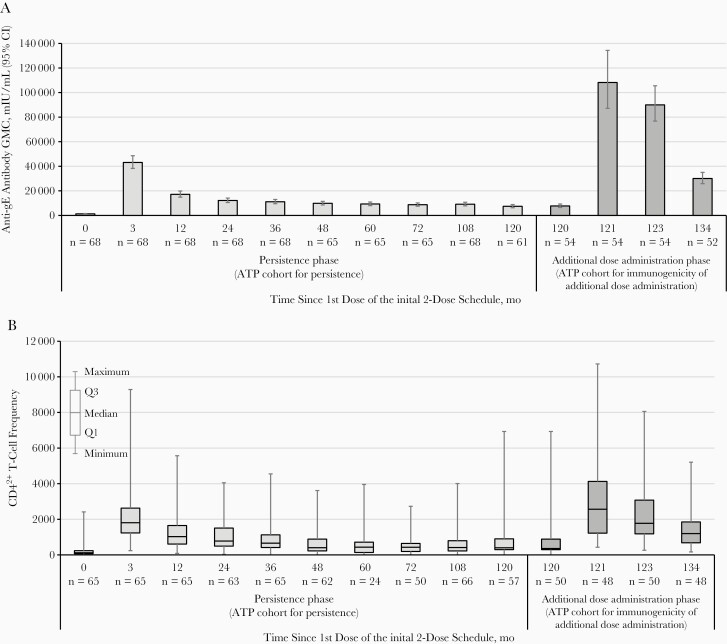
Humoral and cell-mediated immune responses up to 10 years after receipt of the initial 2 doses of the adjuvanted recombinant zoster vaccine (RZV) and in the additional dose administration phase. *A,* Anti–glycoprotein E (gE) antibody geometric mean concentrations (GMCs). *B,* Frequencies of CD4^2+^ T cells (CD4^+^ T cells expressing ≥2 of 4 assessed activation markers: interferon γ, interleukin 2, tumor necrosis factor α, and CD40 ligand). Data also include assessments made in the initial phase II trial and phase II follow-up study. For the additional dose administration phase, months after initial dose 1 represent the following: month 120, before revaccination; month 121, 1 month after additional dose 1; month 123, 1 month after additional dose 2; and month 134, 12 months after additional dose 2. Abbreviations: ATP, according-to-protocol; CI, confidence interval; mo, months; Q1, first quartile; Q3, third quartile.

The anti-gE antibody GMC was 1240 mIU/mL (95% CI, 1003–1535) before initial vaccination, peaked at 43 100 mIU/mL (95% CI, 38 252–48 564 mIU/mL) at 1 month after initial dose 2, and plateaued from month 48 onward, reaching 9123 mIU/mL (95% CI, 7775–10 704 mIU/mL) and 7384 mIU/mL (95% CI, 6203–8790 mIU/mL) at months 108 and 120, respectively (Figure 2A). Descriptive statistics of anti-gE antibody concentrations from before vaccination through month 120 are presented in Supplementary Figure 1*A*.

The median CD4^2+^ T-cell frequency was 121 (interquartile range [IQR], 69–237) before initial vaccination, peaked at 1809 (1233–2627) at 1 month after initial dose 2, and plateaued from month 48 onward, reaching 414 (221–797 [8]) and 402 (298–902) at months 108 and 120, respectively (Figure 2B). Mean CD4^2+^ T-cell frequencies from before vaccination through month 120 are presented in Supplementary Figure 1*B*.

### Safety of the Initial Vaccination

Safety through month 108 after initial dose 1 has been reported elsewhere [6–8]. From month 108 to month 120 after initial dose 1, no vaccine-related SAEs were reported. During the same period, 1 suspected HZ episode was reported >9 years after initial RZV vaccination. A sample from the participant’s lesions was negative for VZV by polymerase chain reaction, when tested at a local laboratory.

### Persistence of Immune Responses Predicted by the Statistical Models

**Figure 3.**
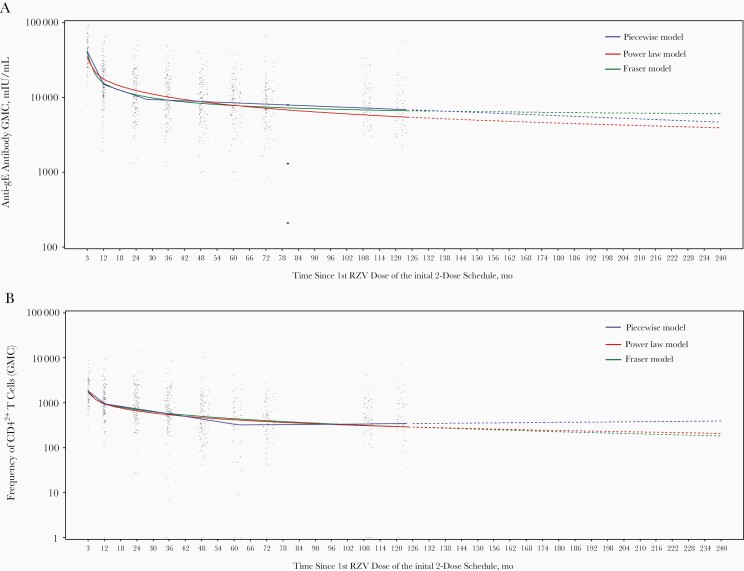
Statistical modeling for prediction of immune responses up to 20 years after receipt of the initial 2 adjuvanted recombinant zoster vaccine (RZV) doses. *A,* Anti–glycoprotein E (gE) antibody geometric mean concentration (GMC). *B*, Geometric mean frequencies of gE-specific CD4^2+^ T cells (CD4^+^ T cells expressing ≥2 of 4 assessed activation markers: interferon γ, interleukin 2, tumor necrosis factor α, and CD40 ligand). Note: Before vaccination, the arithmetic mean CD4^2+^ T-cell frequency was 214.83, and the anti-gE antibody GMC was 1240.

All 3 models based on observations up to month 120 indicate that both anti-gE antibody GMCs (Figure 3A) and geometric mean CD4^2+^ T-cell frequencies (Figure 3B) will remain above prevaccination levels ≥20 years after initial vaccination.

### Immunogenicity of the Additional Doses

The anti-gE antibody GMC increased from 7706 mIU/mL (95% CI, 6423–9246 mIU/mL) before additional dose administration (month 120) to 108 200 mIU/mL (95% CI, 87 154–134 327 mIU/mL) 1 month after additional dose 1 (month 121). No further increase occurred after the second additional dose, with an anti-gE antibody GMC of 90 004 mIU/mL (95% CI, 76 754–105 540 mIU/mL) observed 1 month after additional dose 2 (month 123). The anti-gE antibody GMC persisted above the level before revaccination, up to 12 months after the additional dose 2 (month 134), at 30 066 mIU/mL (95% CI, 25 810–35 024 mIU/mL) (Figure 2A).

The median (IQR) CD4^2+^ T-cell frequency increased from 360 (295–885) before additional dose administration (month 120) to 2564 (1220–4127) 1 month after additional dose 1 (month 121). No further increase occurred after the second additional dose, with a median (IQR) CD4^2+^ T-cell frequency of 1776 (1181–3078) observed 1 month after additional dose 2 (month 123). The median CD4^2+^ (IQR) T-cell frequency persisted above the level before additional dose administration, up to 12 months after the additional dose 2 (month 134), at 1196 (681–1851) (Figure 2B).

### Reactogenicity and Safety of the Additional Doses

**Figure 4.**
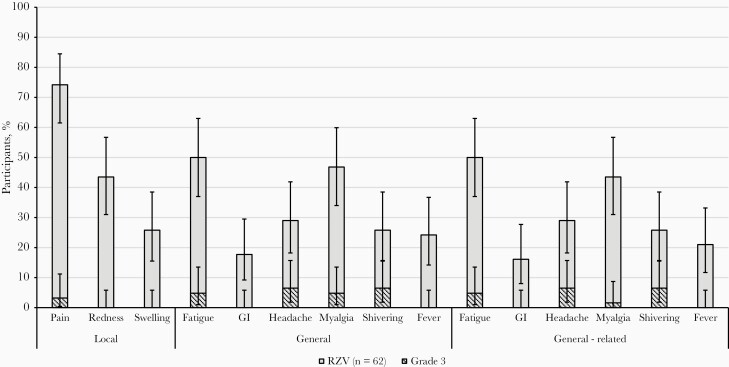
Reactogenicity of the 2 additional adjuvanted recombinant zoster vaccine (RZV) doses. Abbreviation: GI, gastrointestinal symptoms.

The most frequent solicited local AE was pain, reported by 46 RZV recipients (74.2%). Grade 3 pain was reported by 2 RZV recipients (3.2%). The most frequently reported solicited general symptoms were fatigue and myalgia, reported by 31 (50.0%) and 29 (46.8%) of the RZV recipients, respectively. Grade 3 fatigue and myalgia were each reported by 3 (4.8%) participants (Figure 4). Overall, no increase was observed between additional doses 1 and 2 in the frequency of solicited local or general AEs.

Within 30 days of additional dose administration, unsolicited AEs were reported by 14 participants (22.6%; 95% CI, 12.9%–35.0%), of whom 5 (8.1%; 95% CI, 2.7%–17.8%) reported AEs considered by the investigator as vaccine related and 1 (1.6%; 95% CI, .0%–8.7%) reported an AE of grade 3 intensity, which was not considered vaccine related. Most of the vaccine-related unsolicited AEs could be linked to reactogenicity (ie, injection-site reactions and gastrointestinal disturbance). From the first additional dose 1 through study end, 7 participants reported SAEs, none of which were considered vaccine related and none of which were fatal. No potential immune-mediated diseases or suspected HZ episodes were reported in the additional dose administration phase.

## DISCUSSION

In adults vaccinated with 2 initial RZV doses at ≥60 years of age, the humoral and CMI responses persisted through 10 years after vaccination and are predicted to persist ≥20 years after initial vaccination. One additional RZV dose elicited strong humoral and cell-mediated gE-specific anamnestic immune responses, with no further increases observed after the second additional dose. The safety profile of the 2 additional doses was similar to that of the initial 2-dose vaccination course.

**Figure 5.**
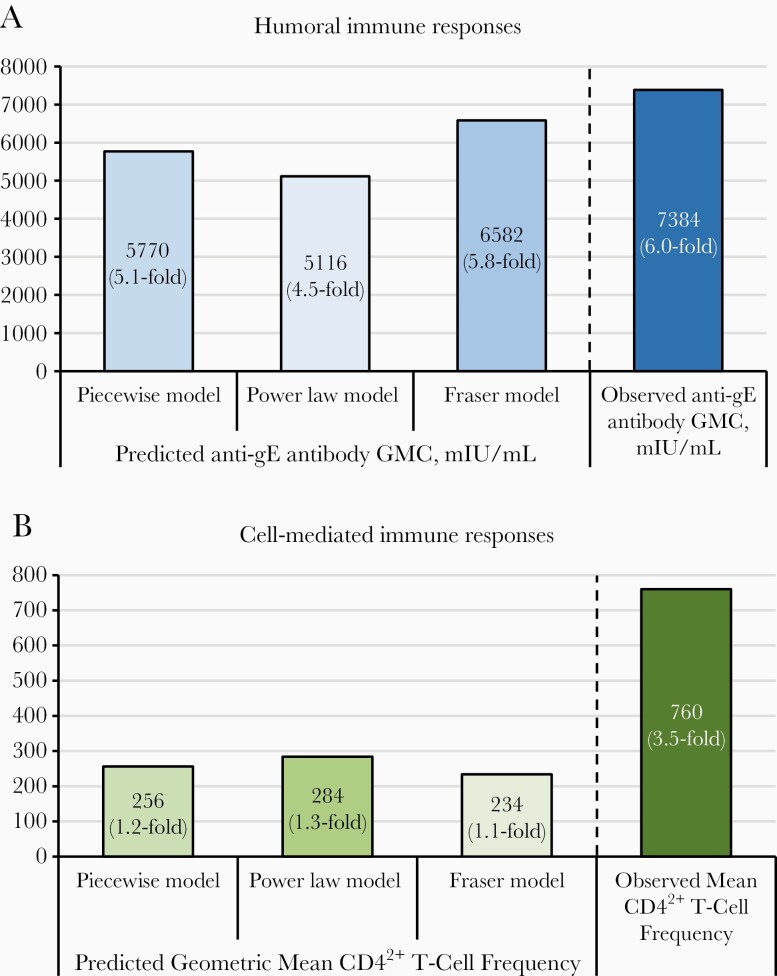
Comparison of predicted versus observed persistence of adjuvanted recombinant zoster vaccine (RZV)–elicited immune responses (including fold increases versus before initial vaccination) 10 years after initial vaccination. *A,* Predicted anti–glycoprotein E (gE) antibody geometric mean concentrations (GMCs), based on data collected through 6 years after vaccination [8, 9]. *B,*Predicted geometric mean frequencies of CD4^2+^ T cells (CD4^+^ T cells expressing ≥2 of 4 assessed activation markers: interferon γ, interleukin 2, tumor necrosis factor α, and CD40 ligand). (These predicted frequencies are presented as fold increases versus the prevaccination arithmetic mean frequency. Because geometric means are always lower than arithmetic means due to the compounding effect, these fold increases are underestimated.)

In participants receiving the licensed RZV formulation in the initial phase II study [6], both humoral and CMI responses have previously been shown to plateau beyond 4 years after initial vaccination [7, 8]. The current data shows that, 10 years after initial vaccination, the anti-gE antibody GMC and the mean gE-specific CD4^2+^ T-cell frequency, respectively, remained 6.0- and 3.5-fold above prevaccination levels. Both of these compare favorably with the predicted levels for 10 years after vaccination using the Piecewise, Power law, or Fraser models, based on observed data through 6 years after vaccination [8, 9] (Figure 5). 

None of these 3 mathematical models overestimated the observed humoral or CMI responses at 10 years after vaccination, confirming the validity of the models used, but there are 2 points for consideration. First, the immune responses 10 years after vaccination were evaluated in the ATP cohort, whereas the predictions were based on the modeling cohort. This should have minimal impact on the observed values, however. Second, for the CMI response, predicted values represent geometric mean CD4^2+^ T-cell frequencies [8, 9], whereas observed values represent arithmetic mean CD4^2+^ T-cell frequencies. Geometric means are always lower than arithmetic means owing to the compounding effect, which could contribute to the approximately 3-fold difference between the observed and the predicted CMI responses (Figure 5).

The Piecewise, Power law, and Fraser models have been previously used to model immune persistence for the 20 years after immunization of young women with the AS04-adjuvanted cervical cancer vaccine [10]. Input of longer-term data improved the profile of the predicted antibody response [10], confirming our observation that these models are conservative when predicting persistence of humoral and CMI responses to adjuvanted vaccines. 

To predict immune response persistence through 20 years after the initial RZV course, the same 3 validated models were used, along with data collected through 10 years after vaccination. All 3 models yielded similar results, indicating that immune responses are predicted to persist above prevaccination levels for ≥20 years. The observed persistence of the immune response after an initial 2-dose schedule observed up to 10 years and predicted to persist up to ≥20 years after initial vaccination, along with the confirmed validity of the prediction models used, suggest that immune responses elicited by the vaccine persist for many years in older adults. These include CMI responses, which are considered the main mechanistic driver of protection against HZ [11]. Data from a large efficacy trial with RZV in adults aged ≥70 years show a high and persistent (84.7%–97.6%) efficacy against HZ during 4 years of follow-up [4], after which both the humoral and CMI responses to RZV plateau [7, 8]. Long-term efficacy data (NCT02723773) and real-world effectiveness data that are being generated for RZV will be needed to confirm the clinical significance of the immune persistence.

Between 9 and 10 years after initial vaccination, no vaccine-related SAEs were reported. During the current study and the previous follow-up study, covering the period between 4 and 6 years, 6 and 9 years, and up to 10 years after initial vaccination, suspected HZ cases were reported in 2 of  ≥70 participants. One case had polymerase chain reaction testing, and results proved negative for VZV; the other had no such testing. Neither case was assessed by an adjudication committee, and HZ cases were not actively sought in these studies.

The ability of a vaccine to induce an anamnestic response is reflected by a rapid and strong immune response elicited in an individual with a previously developed primary immune response to the vaccine’s antigen(s). To assess the ability of RZV to induce anamnestic immune responses in previous RZV recipients, 2 additional doses were administered 2 months apart, 10 years after the initial 2-dose vaccination course. This also allowed assessment of the safety of repeated RZV administration. Both the anti-gE antibody GMC and the median CD4^2+^ T-cell frequency increased substantially after the first additional dose, to higher levels than those elicited by the second dose of the initial vaccination course, revealing the ability of RZV to induce strong anamnestic immune responses. By contrast to the initial 2-dose schedule [6], for which humoral and CMI responses were higher after dose 2 than after dose 1, no further increases were observed after the second additional dose. Hence, the ability of RZV to induce strong anamnestic immune responses was shown after a single additional dose. 

The persistence of immune responses through 1 year after the additional vaccination course was higher for the anti-gE antibody GMCs and similar for the median CD4^2+^ T-cell frequency compared with the initial vaccination course. Long-term efficacy (NCT02723773) and real-world data will enable further assessment of the duration of protection offered by RZV and could help determine the need and optimal timing for additional dosing. The long-term follow-up and revaccination study (NCT02723773), which is currently ongoing, also aims to compare revaccination with 1 or 2 RZV doses. These future data may help us understand whether the anamnestic response induced by the first additional dose reaches a maximum that cannot be further increased by a second additional dose given 2 months after the first additional dose, potentially owing to homeostatic control mechanisms.

The 2 additional RZV doses administered 10 years after the initial 2-dose vaccination course were well tolerated, and no safety concerns were identified. The most frequent solicited AEs after the additional RZV doses were injection-site pain, fatigue, and myalgia. This is in line with the reactogenicity profile of the 2 initial RZV doses previously observed in nearly 10 000 adults ≥50 years of age who participated in the ZOE-50 and ZOE-70 efficacy trials with RZV [12].

**Figure 6.**
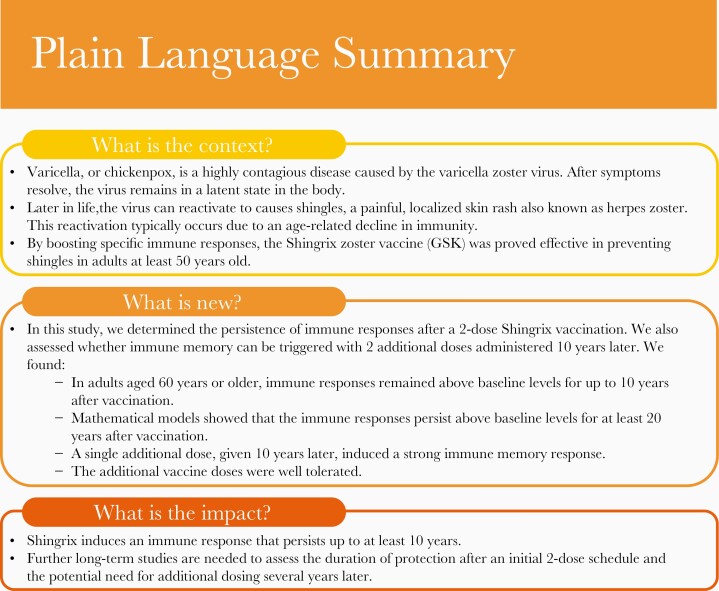
Plain language summary of study results and potential relevance for clinical research.

Interpretation of the data requires consideration of the study limitations. First, although no control group was used in this study, reactogenicity after the initial vaccination may be used as a control for reactogenicity after administration of additional doses. Second, individuals with immunosuppressive conditions or treatment were excluded from participation. However, the mean age of participants was >82 years, thus resulting in a study population considered immunosenescent. Owing to the selection process over time per study design (eligibility criteria, willingness to participate, and medical conditions in an elderly population), the study population may not be fully representative of the vaccinated study cohort enrolled in the initial phase II trial. Despite this general limitation of long-term follow-up studies, almost half of the RZV recipients from the initial phase II study could be enrolled in the long-term follow-up study, and the sample size was adequate to evaluate the study objectives. A plain language summary contextualizing the results and potential clinical research relevance and impact is presented in Figure 6.

In conclusion, in older adults vaccinated at ≥60 years of age, humoral and cellular immune responses induced by 2 initial RZV doses persisted 6.0- and 3.5-fold above prevaccination levels, up to the latest assessment completed in this long-term follow-up study, that is, 10 years after initial vaccination. Statistical modeling predicts persistence of humoral and CMI responses for ≥20 years after initial vaccination. In a population with a mean age of >82 years, additional dosing 10 years after initial vaccination induced strong vaccine-specific anamnestic immune responses.

## Supplementary Data

Supplementary materials are available at *The Journal of Infectious Diseases* online. Consisting of data provided by the authors to benefit the reader, the posted materials are not copyedited and are the sole responsibility of the authors, so questions or comments should be addressed to the corresponding author.

jiaa300_suppl_Supplementary_Material

## Notes

***Acknowledgments.*** The authors thank all study participants, the clinical investigators and study nurses, and the entire GSK study staff involved in the NCT02735915 trial. The authors also thank the Modis platform, on behalf of GSK, for editorial assistance and manuscript coordination. Alpár Pöllnitz provided medical writing support, and Sander Hulsmans coordinated the manuscript development and provided editorial support.

***Author contributions.*** Conceptualization: S. V., C. H., and A. B. Methodology: G. C. Validation: A. H., G. C., A. E., T. M., B. S., S. V., J. S., T. S., C. H., A. B., and A. S. Formal analysis: G. C. and A. E. Investigation: B. S., J. S., L. R., T. S., and K. P. Resources: G. C., A. E., B. S., J. S., L. R., T. S., and K. P. Writing (original draft): A. H., G. C., A. E., C. H., A. B., and A. S. Visualization: A. H., A. B., and A. S. Supervision: T. M., S. V., A. B., and A. S. All authors contributed to the writing (review and editing) of the manuscript and approved the final submission version.

***Financial support.*** This work was supported by GlaxoSmithKline Biologicals SA.

***Potential conflicts of interest.*** A. H., G. C., A. E., T. M., B. S., S. V., C. H., A. B., and A. S. were employees of the GSK group of companies at the time the study was designed, initiated, and/or conducted. C. H. and A. B. are currently an employee of UCB Pharma and Mithra Pharmaceuticals, respectively. A. H., T. M., B. S., A. B., and A. S. hold shares in the GSK group of companies as part of their employee remuneration. T. S. received personal fees from GSK, Pfizer, Biogen, and Merck Sharp & Dohme, outside the submitted work. J. S. reports personal and lecture fees outside the submitted work from GSK, Pfizer, and Sanofi, respectively. L. R. reports lecture fees outside the submitted work from GSK, Pfizer, Sanofi, and Taneka. K. P. certifies no potential conflicts of interest. All authors have submitted the ICMJE Form for Disclosure of Potential Conflicts of Interest. Conflicts that the editors consider relevant to the content of the manuscript have been disclosed.

## References

[1] Yawn BP , Gilden D. The global epidemiology of herpes zoster. Neurology 2013; 81:928–30. 23999562 10.1212/WNL.0b013e3182a3516e PMC3885217

[2] Kawai K , Yawn BP. Risk factors for herpes zoster: a systematic review and meta-analysis. Mayo Clin Proc 2017; 92:1806–21. 29202939 10.1016/j.mayocp.2017.10.009

[3] Lal H , Cunningham AL, Godeaux O, et al; ZOE-50 Study Group. Efficacy of an adjuvanted herpes zoster subunit vaccine in older adults. N Engl J Med 2015; 372:2087–96. 25916341 10.1056/NEJMoa1501184

[4] Cunningham AL , Lal H, Kovac M, et al; ZOE-70 Study Group. Efficacy of the herpes zoster subunit vaccine in adults 70 years of age or older. N Engl J Med 2016; 375:1019–32. 27626517 10.1056/NEJMoa1603800

[5] Cunningham AL , Heineman TC, Lal H, et al; ZOE-50/70 Study Group. Immune responses to a recombinant glycoprotein E herpes zoster vaccine in adults aged 50 years or older. J Infect Dis 2018; 217:1750–60. 29529222 10.1093/infdis/jiy095 PMC5946839

[6] Chlibek R , Smetana J, Pauksens K, et al. Safety and immunogenicity of three different formulations of an adjuvanted varicella-zoster virus subunit candidate vaccine in older adults: a phase II, randomized, controlled study. Vaccine 2014; 32:1745–53. 24508036 10.1016/j.vaccine.2014.01.019

[7] Chlibek R , Pauksens K, Rombo L, et al. Long-term immunogenicity and safety of an investigational herpes zoster subunit vaccine in older adults. Vaccine 2016; 34:863–8. 26432913 10.1016/j.vaccine.2015.09.073

[8] Schwarz TF , Volpe S, Catteau G, et al. Persistence of immune response to an adjuvanted varicella-zoster virus subunit vaccine for up to year nine in older adults. Hum Vaccin Immunother 2018; 14:1370–7. 29461919 10.1080/21645515.2018.1442162 PMC6037441

[9] Lal H , Chlibek R, Pauksens K, et al. Persistence of the immune response to an adjuvanted herpes zoster subunit vaccine in healthy older adults: modeling of vaccine-induced immune response, data from a 6-year follow-up study. Open Forum Infect Dis 2015; 2:1931.

[10] David MP , Van Herck K, Hardt K, et al. Long-term persistence of anti-HPV-16 and -18 antibodies induced by vaccination with the AS04-adjuvanted cervical cancer vaccine: modeling of sustained antibody responses. Gynecol Oncol 2009; 115:S1–6. 19217149 10.1016/j.ygyno.2009.01.011

[11] Weinberg A , Levin MJ. VZV T cell-mediated immunity. Curr Top Microbiol Immunol 2010; 342:341–57. 20473790 10.1007/82_2010_31

[12] López-Fauqued M , Campora L, Delannois F, et al; ZOE-50/70 Study Group. Safety profile of the adjuvanted recombinant zoster vaccine: pooled analysis of two large randomised phase 3 trials. Vaccine 2019; 37:2482–93. 30935742 10.1016/j.vaccine.2019.03.043

