# Supplementary material for: Immunogenicity of the Adjuvanted Recombinant Zoster Vaccine: Persistence and Anamnestic Response to Additional Doses Administered 10 Years After Primary Vaccination
Source: J Infect Dis. 2020 Jun 5;224(12):2025–34. doi: 10.1093/infdis/jiaa300 (PMC8672743; doi:10.1093/infdis/jiaa300)
Supplement: jiaa300_suppl_Supplementary_Material [file jiaa300_suppl_supplementary_material.docx]

**Immunogenicity of the adjuvanted recombinant zoster vaccine: persistence and anamnestic response to additional doses administered 10 years after primary vaccination**

# Supplementary material

**Supplemental Text 1.** Inclusion/exclusion criteria

Inclusion criteria:

- Participants who, in the opinion of the investigator, could and would comply with the requirements of the protocol or participants with a caregiver who, in the opinion of the investigator, could and would comply with the requirements of the protocol;
- Written informed consent obtained from the participant prior to performing any study-specific procedure;
- Previous participation in the initial Phase II trial (NCT00434577), in the group receiving the licensed adjuvanted recombinant zoster vaccine (RZV) formulation, and who completed the 2-dose vaccination course;
- Participants who completed visit 1 between 108 and 111 months after the first RZV dose of the previous vaccination course in the initial study (NCT00434577)

Exclusion criteria:

- Use of any investigational or non-registered product other than the study vaccine during the period starting 30 days before the first study visit, or planned use during the study period;
- Use or anticipated use of immunosuppressants or immune-modifying drugs during the period starting 6 months prior to study start and during the whole study period. This included chronic administration of corticosteroids, long-acting immune-modifying agents or immunosuppressive/cytotoxic therapy;
- Any confirmed or suspected immunosuppressive or immunodeficient condition that resulted from disease;
- Administration or planned administration of a live vaccine in the period starting 30 days before the first dose of study vaccine and ending 30 days after the last dose of study vaccine, or, administration or planned administration of a non-replicating vaccine within 8 days prior to or within 14 days after either dose of study vaccine;
- Previous vaccination against herpes zoster since initial vaccination in initial study (NCT00434577);
- Administration of immunoglobulins and/or any blood products during the period starting 3 months before study start, or planned administration during the study period;
- History of herpes zoster

**Supplemental Text 2.** Grading of adverse events

Solicited AEs were graded on a scale from 0 (characterized by a surface diameter <20 mm [for swelling and redness], oral temperature <37.5 ⁰C [for fever] or absent/normal [for all other solicited AEs]) to 3 (characterized by a surface diameter >100 mm [for redness and swelling], significant at rest and preventing normal everyday activities [for pain], body temperature >39.0 ⁰C [for fever] or preventing normal activity [for all other solicited AEs]). Unsolicited AEs were graded by the investigator on a scale from 1 (mild: easily tolerated, causing minimal discomfort and not interfering with everyday activities) to 3 (severe: preventing normal everyday activities).

**Supplemental figure 1.** Humoral and cell-mediated immune responses up to 10 years post-initial vaccination* (ATP cohort for persistence)

A


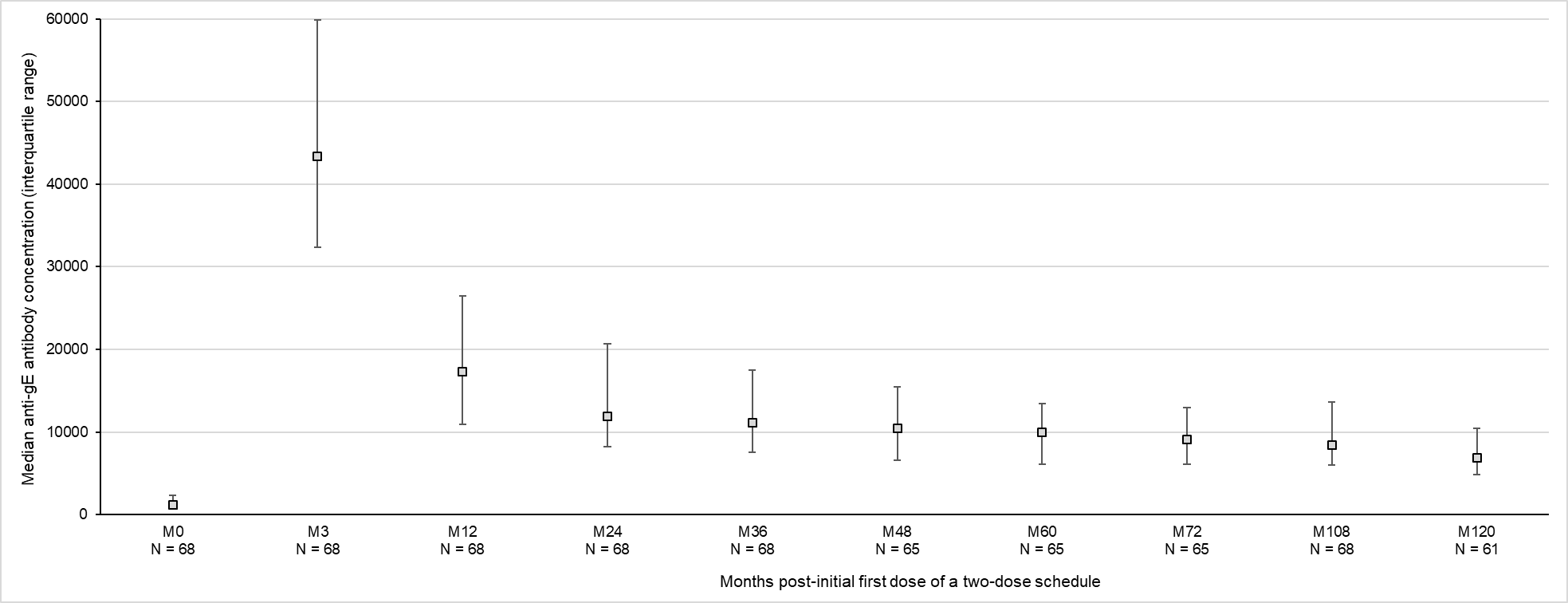


B


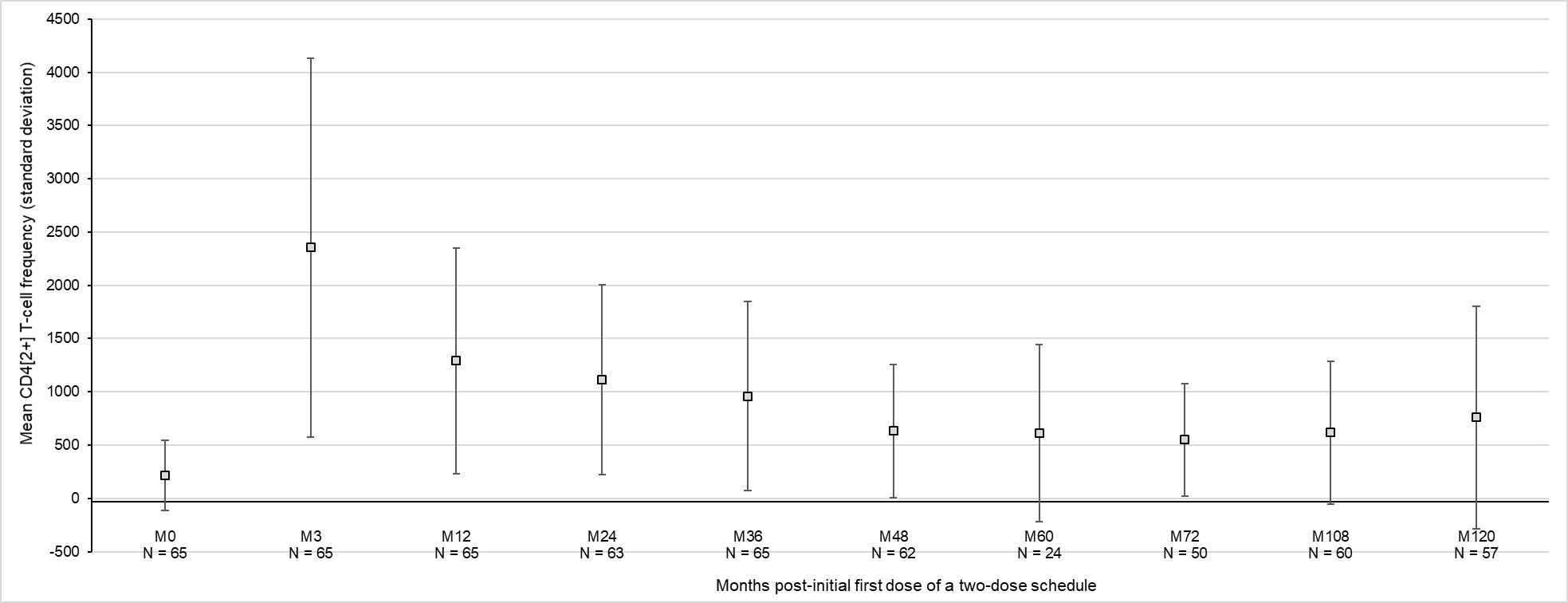


(A) Descriptive statistics of anti-gE antibody concentrations**; (B) Mean frequencies of CD4[2+] T cells

ATP, according-to-protocol ; gE, glycoprotein E; CD4[2+] T-cells, CD4 T-cells expressing at least 2 of 4 assessed activation markers: interferon-γ, interleukin-2, tumor necrosis factor-α and CD40 ligand; N, number of participants with available results; M, month.

*Also includes assessments made in the initial Phase II trial and Phase II follow-up study [[6](#_ENREF_6), [7](#_ENREF_7)].

** Data through M108 have been previously presented previously [[8](#_ENREF_8)] and are also included here for completeness
